# Sammelbesprechung

**DOI:** 10.1007/s00048-022-00326-x

**Published:** 2022-02-10

**Authors:** Annette Imhausen

**Affiliations:** Goethe Universität Frankfurt, Frankfurt am Main, Germany

**Florence Bretelle-Establet and Stéphane Schmitt (eds.) 2018: *Pieces and Parts in Scientific Texts (Why the Sciences of the Ancient World Matter, vol. 1). ***Cham: Springer International Publishing, geb., 355 S., 128.39 €, ISBN: 978-3-319-78466‑3.

**Christine Proust, and John Steele (eds.) 2019: *Scholars and Scholarship in Late Babylonian Uruk (Why the Sciences of the Ancient World Matter, vol. 2). ***Cham: Springer International Publishing, geb., 274 S., 24 s/w Abb., 128.39 €, ISBN: 978-3-030-04175‑5.

**Cécile Michel and Karine Chemla (eds.) 2020: *Mathematics, Administrative and Economic Activities in Ancient Worlds (Why the Sciences of the Ancient World Matter, vol. 5). ***Cham: Springer International Publishing, geb., 568 S., 127 s/w Abb., 35 farb. Abb., 117.69 €, ISBN: 978-3-030-48388‑3.

In early accounts of the history of science, research about ancient sciences was often limited to the role of providing the starting points of their respective modern successors. Historians of science working in these areas were mainly appreciated as those who could read texts written in strange languages using obscure scripts. As has been documented since, this perspective developed as a result of the subjects chronologically early position in the development of scientific knowledge but also due to a Eurocentric bias that privileged European developments over those elsewhere. Over time and as a result of the successful research of historians of ancient sciences, however, it became obvious that the ancient sciences are not just rudimentary elements of modern sciences that developed in a straightforward way from then to now. Rather, each time and location has developed its own scientific concepts, forms and practices, and in fact one need not go back very far to notice significant differences between what was considered science in an earlier period and today. The historiographic development towards this insight was achieved gradually, and its implications continue to stimulate new research. With it also came a gradual appreciation of the actual work of historians of the ancient sciences beyond their mere abilities as translators.

The new series *Why the Sciences of the Ancient World Matter* published by Springer carries the recently acquired self-confidence of the ancient history of science in its title. It not only implies that ancient sciences need to be studied in their own right, but also that the history of later sciences and those interested in the development of sciences may profit from the results of studying scholars, learned texts, and scholarly practices in ancient times. This review examines three volumes (1, 2, and 5) of that series, which aims to provide “a platform for the publication of studies on sciences in the ancient worlds that bring innovative methods into play and address new theoretical issues” (vol. 1: ii).

The first volume, *Pieces and Parts in Scientific Texts*, is, judging from its title, the most general of the three volumes reviewed here. The table of contents, however, reveals that following the first two introductory chapters, there is a certain focus on mathematics, which features in three out of ten chapters (chapters 3, 5, 6). Other sections cover medicine (chapters 7, and 8), astronomy (chapter 4), and natural history (chapter 9). Only the final chapter “Collecting Languages, Alphabets and Texts: The Circulation of ‘Parts of Texts’ Among Paper Cabinets of Linguistic Curiosities (Sixteenth-Seventeenth Century)” focuses on a philological subject. Having worked in several projects (some of which focus on a much larger scope than volume 1 of the series) that attempted to present a multi-geographical and trans-epochal perspective, I am aware of the immanent difficulties of projects of this kind: not all regions and times provide suitable material for a successful study of the proposed topic, and secondly, finding a scholar willing and able to take part in a project of this kind can be equally challenging. Therefore, criticism of the volume for not including this or that place and time is rather trivial. However, the selection found in the first volume might have benefitted from restricting itself to the ancient period and including other ancient cultures that seem often left out (e.g. ancient Egypt and ancient Rome). For a “different” perspective on texts, Maya inscriptions might have been an interesting topic to include. On the other hand, a volume that combines ancient Mesopotamia, Greece, China, India, and early modern Europe seems promising for its inclusion of a variety of scientific texts with different characteristics. In the 19th and early 20th century, the history of ancient science was often undertaken anachronistically from a modern perspective by searching for scientific elements in ancient texts that could be linked to their modern successors. In the meantime, many scholars have pointed out the flaws of this approach as well as the Eurocentric bias that has led to the neglect of ancient non-western scholarship (e.g. *The History of Mathematical Proof in Ancient Traditions* edited by Karine Chemla or Francesca Rochberg’s *Before Nature*). In this volume, the choice of the second chapter (meant to provide a framework for the various studies on ancient texts) on “parts of texts” based on modern European sources seems a little awkward and runs counter to the explicitly stated aspiration to execute a global, multi-epochal project focused on antiquity. Despite this criticism, the individual chapters are each interesting studies that present the selected material and make use of specific features of the sources (e.g. structural organization of mathematical and astronomical texts from Mesopotamia, Greece, India and China) that do not fall into the trap of an anachronistic Eurocentric perspective.

*Scholars and Scholarship in Late Babylonian Uruk* (vol. 2 of the series) focuses on scholarly texts from Uruk originating from the second half of the first millennium BCE. The individual contributions include an introduction into the setting of scholarly Uruk followed by an article on commentaries (chapter 2), two chapters on mathematics (chapters 3 and 6) and three chapters on the celestial sciences (chapters 4, 5, and 7). The final two contributions analyze the relation between Uruk and the Greco-Roman World (chapters 8 and 9). John Steele discusses a group of forty astronomical-astrological tablets that were discovered in the house of āšipus (medical practitioner, formerly also translated as exorcist) which indicate the many uses of celestial sciences by this group of scholars. The texts include more astrological (i.e. texts that relate astronomical data to information about the human realm) than astronomical (i.e. texts on descriptions, observations or calculations of celestial phenomena). The astronomical texts are described in detail and provide insight into the level of technical astronomical texts that the āšipus apparently used. Mathieu Ossendrijver focuses on the location of the Rēš temple in Uruk, compiling and analyzing mathematical sources from its scholars and linking them to earlier libraries in Uruk. Julia Krul looks at the relation of astral sciences and temple rituals in Hellenistic Uruk and Babylon, providing the means for an appreciation of the relevance of astral sciences in the context of the daily life of a temple. The volume thus achieves a quite detailed picture concerning mathematics and astronomy-astrology in Late Babylonian Uruk. It would have been interesting to see a picture that also includes other scholarly areas that can presumably be found within the Uruk material as well, for example by the inclusion of other tablets from the house of āšipus, to match the broad scope of the title given to the volume—however, that would have required a larger workshop beforehand that would probably not have corresponded as well with the ERC project from which this volume results.

Volume 5, *Mathematics, Administrative and Economic Activities in Ancient Worlds* fills a longstanding need to situate mathematics into its context of administration in which it originated and developed in various societies. This volume, too, follows a multicultural approach with several examples from Mesopotamia (Cécile Michel, Stephanie Rost, Martin Sauvage, Camille Lecompte, Christine Proust, Robert Middeke-Conlin), India (Mark McClish, Sreeramula Rajeswara Sarma and Takanori Kusuba), China (Hao Peng, Karine Chemla and Biao Ma), and a final contribution from medieval France (Marc Bompaire and Matthieu Husson). Egypt, which has few mathematical texts but a wealth of sources from accounting, could have made a valuable contribution to this volume, but was, unfortunately, not included. The volume is organized in four parts that—at least to the uninitiated reader—provide less of a structural frame than one might assume from their titles. Thus “Quantifying Spatial Extension, Quantifying Work,” as the second part is called, might well have included Part III “Quantifying Land and Surfaces.” Some of the contributions attempt to combine sources from the mathematical and administrative realms, while others focus on the mathematics in administrative texts. The volume compensates for this lack of coherence of the individual contributions through the wealth of possible lines of inquiry it opens up that may pursued systematically in various locations and times by other scholars.

Judging from the three volumes under review here, as well as the title of two further volumes (*Monographs in Tang Official Historiography* (vol. 3) edited by Daniel Patrick Morgan and Damien Chaussende and *The Making of a Scribe* (vol. 4) by Robert Middeke-Conlin) the series is representative of current research in ancient sciences. As such, it indicates its current potential and speaks to the better understanding that is to be gained from appreciating the setting of scholarly developments like administration for mathematics and the ritual context for astral sciences that result from the development of history of ancient sciences over the last decades. It is worth noting that this series is not the only new series on ancient sciences that has emerged in the last few years. The online publications by the Institute for Research in Classical Philosophy and Science and the new series *Ancient Cultures of Sciences and Knowledge* are two further examples that focus explicitly on this research area. Taken together, these publications attest to the lively and active community of historians of science working on ancient sources and the potential to learn about the origin and early development of sciences and their place within societies which—judging by recent developments—has become a point of concern in many parts of the world.

